# 3Rs missing: animal research without scientific value is unethical

**DOI:** 10.1136/bmjos-2018-000048

**Published:** 2019-07-04

**Authors:** Daniel Strech, Ulrich Dirnagl

**Affiliations:** 1 QUEST Center for Transforming Biomedical Research, Berlin Institute of Health (BIH), Berlin, Germany; 2 Charité - Universitätsmedizin Berlin, Berlin, Germany; 3 NeuroCure Clinical Research Center, Charité - Universitätsmedizin Berlin, Berlin, Germany

## Abstract

The current, widely established 3R framework for the ethical use of animals in research consists of three guiding principles, that is, *R*eplacement, *R*eduction and *R*efinement, all aiming to safeguard the overarching ethical principle of animal welfare. However, animal welfare alone does not suffice to make animal research ethical if the research does not have sufficient scientific value. The scientific value of animal studies strongly decreases if they are not sufficiently robust, if their questions have already been sufficiently addressed or if the results are selectively reported. Against this background, we argue that three guiding principles are missing, that is, *R*obustness, *R*egistration and *R*eporting, all of which aim to safeguard and increase the scientific value of animal research. To establish a new 6R framework, we need a multistakeholder discourse to conceptualise the specific requirements of robustness, registration and reporting and to clarify responsibilities, competencies and legislation for auditing 6R compliance.

## Introduction

Framed by Russel and Burch more than 60 years ago, the 3Rs (*R*eplacement, *R*eduction and *R*efinement) have become the guiding principles for the ethical use of animals in research.^1^ Although universally accepted, there is an ongoing discourse on their improvement, uptake and implementation.^2^ Here, we argue that with their current focus on animal welfare, the 3Rs lack an important ethical dimension. Research on animals is only ethical if it generates value for science and society, a dimension that is not represented by the current 3Rs.

Individual research projects are only valuable if they enable a knowledge gain, apply robust study designs and report their results in a non-selective manner. Whether a research project will ultimately contribute to innovation in healthcare is hard to gauge for several reasons. One reason is that scientific breakthroughs may take years to manifest. Robustness, on the other hand, can be judged on the research project level. If we want to better understand what research questions are still insufficiently addressed, we need individual projects to be accessible via animal study registries open to the public. Furthermore, only if protocols are prospectively registered are we able to identify selective reporting of study results.

**Figure 1:**
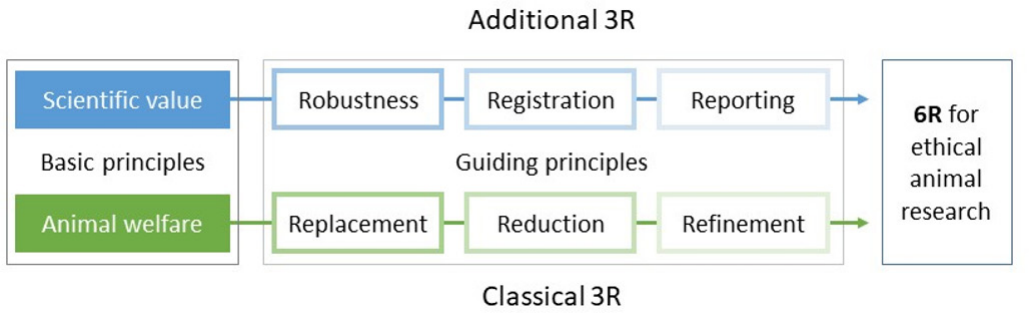
Two basic principles for animal research ethics translate into six practice-guiding principles (6R).

We posit that while the current 3Rs are important for upholding animal welfare, the dimension of scientific value needs to be considered when planning, reviewing and conducting animal research. We therefore propose the addition of three additional Rs, that is, *R*obustness, *R*egistration and *R*eporting, to the guiding principles for the ethical use of animals in research (figure 1).

## Why do we need to complement the 3R framework now?

Over the past 5 years, several empirical studies and expert analyses have demonstrated that three challenges endanger the value of animal research. First, animal research often lacks measures to reduce validity threats such as biases or a lack of statistical power.^3 4^ Second, animal research faces a substantial publication bias, that is, null and negative results often end up in the file drawer.^5 6^ Third, publication of results often lacks important information that is needed for a critical appraisal (eg, information on study design or attrition of animals).^7 8^ These challenges negatively affect the reproducibility of animal studies^9 10^ and the relevance of animal studies in justifying early human research.^11 12^ In summary, these threats reduce the value of the research results, potentially leading to inefficient allocation of public funds, to ill-advised clinical research and to the unnecessary use and suffering of experimental animals.

## Why robustness, registration and reporting?

Our core argument is that the current 3R principles for animal research, despite their importance, are limited because of their one-sided focus on the basic ethical principle ‘animal welfare’. They lack an explicit and practice-oriented set of guiding principles promoting the second basic ethical principle ‘scientific value’. Furthermore, each of the additional 3R principles (robustness, registration and reporting) is important in itself and not replaceable by the other two. Animal studies, for example, can be robust but reported in a biased or otherwise inappropriate way. Alternatively, they can be appropriately reported but not robust. Both scenarios compromise the value of the study. In times where approximately 50% of animal studies are not reported,^13^ only the preregistration of animal study protocols allows the identification of biased, delayed or unreported results. Finally, ethics frameworks for human research already address all three value principles for the same moral reasons. The Declaration of Helsinki, for example, includes registration (article 35) and reporting (article 36) as obligatory principles.^14^ The widely acknowledged framework for clinical research ‘What makes clinical research ethical’ from Emanuel *et al* highlights robustness (scientific validity) as one of the basic ethical principles.^15^

## How do we implement the new 3Rs in current practices?

The reporting principle is relatively easy to implement. Beside standard peer-review journals, new publication formats allow accessible reporting of all types of research results, including null and negative results, such as preprint servers (eg, bioRxiv), Open Access journals (eg, BMJ Open Science, PLoS One), journals with postpublication review (eg, f1000research) or data repositories (eg, Open Science Framework, Dryad, figshare). Adherence to reporting guidelines, such as ARRIVE,^7^ further aims to improve the evaluation and utilisation of study results. Several leading research funders such as the Wellcome Trust, the Horizon 2020 programme or the Bill and Melinda Gates Foundation just recently signed the WHO Joint Statement and thus indicated to make reporting requirements a part of funding decisions for clinical trials.^16^ Similarly, ethics review and funding of individual animal studies could implement a requirement for timely and non-selective results reporting and evaluate compliance.

Dedicated tools for implementing the registration principle in animal research equivalent to registries for human studies (such as ClinicalTrials.gov) have already been launched by academic initiatives (eg, www.preclinicaltrials.eu) or just recently by a governmental organisation (www.animalstudyregistry.org). These platforms allow swift protocol registration with an embargoing option for several years. The registration principle will increase the value of research but how will it affect the efficiency of animal research? In a recent study, experts from all relevant stakeholder groups in animal research expressed their attitudes on potential strengths and weaknesses of animal study registries.^17^ Some highlighted their concerns that animal study registration might aggravate administrative burdens and the theft of ideas. Others emphasised the opposite viewpoint that improved transparency via such registries might ultimately make animal research more efficient.

The robustness principle is more difficult to implement: How can we gauge robustness of individual animal studies? More specifically: When is sample size calculation or blinded outcome assessment necessary? How can the external and construct validity of individual studies be improved? Recent expert proposals to better distinguish between exploratory and confirmatory study designs in animal research have provided preliminary answers.^18 19^ Initial guidance on how to implement a more systematic assessment of animal study robustness in standard review procedures was recently published by Würbel.^20^ Würbel distinguishes three dimensions of validity (internal, external and construct validity) and recommends assessing each dimension within the harm–benefit analysis for individual animal studies. With this proposal, he is in line with recent guidance from Kimmelman on how to assess the validity of animal studies within approval procedures for phase I/II clinical trials.^21^ Assessing robustness of individual studies requires complex judgements. Ethics review boards for animal studies, however, already require complex judgements regarding the welfare principles, and in many jurisdictions, already consider a study’s robustness. Even Russel and Burch already included a section on ‘The Design and Analysis of Experiments’ in the chapter explaining the Reduction principle.^1^ They emphasise the importance of statistics to determine the minimum number of animals needed for an experiment and they mention sequential analysis and randomisation as further means to reduce uncontrolled variance. They do not emphasise, however, robustness or scientific value as a principle in itself and they do not mention further measures to improve robustness such as blinding of outcome assessment.

In line with our recommendation to add guiding principles for scientific value to the ethical framework for animal research are recent activities from national centres for the 3Rs such as the UK National Centre for the Replacement Refinement & Reduction of Animals in Research (NC3Rs) or the German Centre for the Protection of Animals in Research (Bf3R). Both already promote the new 3R principles for scientific value in several ways. The revised NC3Rs guidelines for primate research, for example, explicitly require robustness and reporting.^22^ The new NC3Rs Experimental Design Assistant (EDA) not only supports the development of robust study protocols but also allows to timestamp the resulting protocols. With the option to make such timestamped protocols publicly available, the EDA facilitates preregistration of protocols on a voluntary basis.^23^ In January 2019, Bf3R launched their Animal Study Registry. We very much welcome these recent developments but want to highlight that they do not derive directly from any of the three animal welfare principles. They make sense only when considering scientific value as a complementary set of ethical principles.

## ‘Rhumba of Rs’?

In the previous sections, we already commented on potential counterarguments against the introduction of a complementary set of 3R principles. These counterarguments addressed the relevance or implementability of registration, robustness, or reporting in a direct way. Another type of counterargument is more indirect: Does it make sense at all to add new R principles? At least two arguments were raised in our discussions with colleagues and reviewers: first, other papers already and unsuccessfully proposed new Rs such as Responsibility, Reproducibility or Rigour. These contributions did not impact on animal research but rather heat up a rhumba of Rs.^24^ We think that former proposals of new Rs were unsuccessful because they were circular, too broad, or did not provide direct guidance. Responsibility as an R principle is clearly circular, as it cannot specify how to act responsibly. Reproducibility as an R principle does not provide direct guidance. It is a desired characteristic of animal research that strongly depends on robustness and non-selective reporting. Rigour as an R principle is too broad, at least in its current use. Rigour is often used interchangeably with scientific value as it comprises robustness, non-selective reporting and could also comprise registration.

The second counterargument against any modification of the 3R framework is based on the assumption that the current 3R framework is a strong concept especially because it is established all over the world. Adding new Rs bears the risk to dilute this widely accepted concept, ultimately leading to a weaker protection of animal welfare. However, we do not find it plausible to believe that a consistent set of three new guiding principles that all centre around the complementary basic principle of scientific value will dilute the very distinct basic principle of animal welfare. In contrast, we posit that the relatively narrow focus of the current 3R approach contributed to the fact that animal research often lacks scientific value.

## Summary

Animal research is ethical only when it is of scientific and social value. The past years have demonstrated that this value of animal research and thus its capacity to improve human health are threatened by a lack of robustness and biased or unreported results. Three ethical principles (*R*obustness, *R*egistration and *R*eporting) help to safeguard the value of animal research. The current, widely established ethical framework for animal research (3Rs=*R*eplacement, *R*eduction and *R*efinement) misses this value dimension by solely focusing on the equally important animal welfare dimension. We recommend complementing the current 3R framework (for animal welfare) with the second set of 3Rs (for scientific value). Regulators, ethics boards, scientists and funders should add robustness, registration and reporting to their criteria when planning, licensing or funding animal experiments. Guidances such as the Basel Declaration should consider making the normative framework for animal research more comprehensive and coherent.^25^ National centres for the 3R should consider revising their branding and explicitly addressing the ethical rationale underlying their recent policies for registration, robustness and reporting. To this end, a multistakeholder discourse and decisions are needed to (1) conceptualise the specifics of robustness, (2) develop frameworks detailing the mandatory information that is being registered as well as acceptable embargo periods, (3) clarify funding and approval requirements related to results reporting and (4) determine relevant responsibilities, competencies and legislation for auditing 6R compliance.
